# Potential US Health Care Savings Based on Clinician Views of Feasible Site-of-Care Shifts

**DOI:** 10.1001/jamanetworkopen.2024.26857

**Published:** 2024-08-14

**Authors:** Nikhil R. Sahni, Crosbie Marine, David M. Cutler, Laura N. Medford-Davis, Melvin Mezue, Omar Kattan, Ed Levine, Karen E. Joynt Maddox

**Affiliations:** 1 Department of Economics, Harvard University, Cambridge, Massachusetts; 2 Center for US Healthcare Improvement, McKinsey and Company, Boston, Massachusetts; 3 National Bureau of Economic Research, Cambridge, Massachusetts; 4 Cardiovascular Division, Department of Medicine, Washington University School of Medicine, St Louis, Missouri; 5 Center for Advancing Health Services, Policy and Economics Research, Institute for Public Health, Washington University, St Louis, Missouri

## Abstract

**Importance:**

Shifting care to alternative sites when clinically appropriate may be associated with reduced US health care spending, improved access, and, in some cases, improved care outcomes.

**Objective:**

To fill 2 main gaps in the current literature on site-of-care shifts: (1) understanding the clinician perspective on appropriateness of alternative care sites, given the central role they play in referrals and patient trust and (2) considering all potential sites where care could shift and calculating net savings potential.

**Design, Setting, and Participants:**

In this survey study, physicians (MDs and DOs), nurse practitioners, physician assistants, nurse anesthetists, radiology and imaging technicians, and psychologists were surveyed from September 17 to November 22, 2021, about potential shifts of care from the hospital setting to alternative sites. Participants were selected by the survey firm Intellisurvey to provide broad representation across all specialties of interest. A minimum of 34 clinicians responded to each question. Data were analyzed from April 2022 through October 2023.

**Exposure:**

More than 5000 individual diagnostic and procedural codes were reviewed and sorted into 312 distinct care activities by an expert panel of physicians. Survey respondents were then provided with the 2019 claims-based distribution across sites of care for each care activity and were asked, “based on your clinical judgment, what portion of [care activity] could safely occur in each of the following sites of care, without compromising clinical outcomes?”

**Main Outcomes and Measures:**

Based on clinician-reported distributions, the total potential shift of volume from hospital-based settings to alternative sites and the associated net savings were estimated.

**Results:**

Survey respondents included 1069 practicing clinicians (386 female [36.1%]; mean [SD] years since residency of physicians, 21.0 [9.7] years; mean [SD] age of nonphysicians, 45.3 [9.4] years) across specialties, all of whom practiced more than 20 clinical hours per week. There were 794 physicians (74.3%), and the remaining 275 respondents were midlevel professionals, such as physician assistants. Among 312 care activities surveyed, respondents indicated that 10.3 percentage points (95% CI, 10.0-10.5 percentage points) of commercial and 10.9 percentage points (95% CI, 10.7-11.1 percentage points) of Medicare volume currently taking place in hospital-based settings could shift to alternative sites with today’s technology without compromising clinical outcomes. Across the entire US health care system, these shifts could be associated with a reduction in overall health care consumption spending ($3 562 339 000 000 000) by approximately $113.8 billion ($113 767 446 087 174 [3.2%]) to $147.7 billion ($147 661 672 284 263 [4.1%]) annually.

**Conclusions and relevance:**

In this study, a substantial net savings opportunity was estimated. However, realizing this potential will require ongoing alignment among organizations, clinicians, and policymakers to overcome barriers to these shifts.

## Key Points

**Question:**

Based on clinician views, how much care from the hospital could be shifted to alternative sites without compromising clinical outcomes, and what would be the associated net savings?

**Findings:**

In this survey study of 1069 clinicians surveyed in 2021 and historical claims data from 2019, 10.3 percentage points of commercial and 10.9 percentage points of Medicare volume could be shifted from the hospital to alternative sites using today’s technology without compromising clinical outcomes. Based on observed reimbursement rates, this would be associated with savings of $113.8 billion (3.2%) to $147.7 billion (4.1%) in 2019 dollars annually for the overall US health care system.

**Meaning:**

These findings suggest that while a substantial net savings opportunity may remain from site-of-care shifts, ongoing alignment among organizations, clinicians, and policymakers is needed to ensure the best outcomes for patients.

## Teaser

This survey study assesses clinician views on the feasibility of site-of-care shifts from the hospital to alternative care settings and estimates potential net savings that would be associated with these shifts.

## Introduction

Health care delivery in the US is moving rapidly away from traditional sites of care. Contrasted with hospital-based care, clinically appropriate care provided in nonhospital settings, including urgent care, ambulatory surgery centers (ASCs), postacute facilities, and patient homes, generally costs less, is easier to access, and may be associated with improved care outcomes.^1,2,3,4,5,6,7,8,9,10,11^ In a signal that the business community sees this as an opportunity for innovation and cost savings, the number of venture capital and private equity deals focused on alternative sites more than doubled from 2010 to 2021.^12,13,14^ Recent mergers and acquisitions have also increased in this space, with larger organizations acquiring innovative and often nonhospital sites.^15,16,17,18^

Prior, largely nonacademic research has suggested that $250 billion or more of spending and up to 30% of inpatient bed days could be shifted to alternative sites, such as home or virtual.^19,20,21^ However, these studies are limited in their generalizability because they often represent gross savings not net savings and examine 1 site at a time vs holistically considering all potential sites where care could shift.^1,2,3,4,5,6,7,8,9,10,11^

In addition, current literature has a limited perspective on what care clinicians believe could be shifted without compromising clinical outcomes. Given that clinicians play a central role in guiding patients to specific services and locations and that their clinical insights may give them a unique perspective into the safety and efficacy of proposed shifts, this is an important gap in the literature.

Historically, studies have used survey instruments to gather opinions from populations like clinicians to then extrapolate broader findings for the US health care system.^22,23,24,25^ Given this, we undertook a multispecialty clinician survey with 2 goals. First, we used the results of a survey of more than 1000 clinicians to assess the potential for shifts in site-of-care distributions for more than 300 care activities, today and with expected future technologies, without compromising clinical outcomes. Second, we used 2019 claims data for commercial and Medicare populations to estimate potential net savings associated with these shifts.

## Methods

This survey study was determined to be exempt from institutional review board review because it did not involve the treatment of patients or the use of identifiable private information. The development, execution, analysis, and reporting of this study adhered to the American Association for Public Opinion Research (AAPOR) reporting guideline.

### Survey Development

Full details of survey development are available in the eMethods in Supplement 1. Briefly, we formed an expert panel of 33 physicians to review and sort more than 5000 individual diagnostic and procedural codes into 312 distinct moderate- to high-volume care activities (eg, coronary angiography and percutaneous intervention or ankle surgery). These care activities were then cataloged by primary responsible specialty (eg, cardiology or orthopedic surgery). In aggregate, this represented 1 366 795 126 of 2 802 036 687 activities (48.8%) for commercial volume and 930 847 760 of 1 877 384 400 activities (49.6%) for Medicare fee-for-service volume and $342 915 136 514 of $626 066 204 300 (54.8%) and $197 687 732 351 of $422 044 506 806 (46.8%) of spending, respectively, in our claims sample. For each of 312 care activities, we then calculated the 2019 claims-based distribution across sites of care, which was reviewed for accuracy by the expert clinician panel.

Each of 1069 survey respondents was provided the list of care activities relevant to his or her specialty. When more than 1 specialty might direct the same activity (eg, spine surgery or the care of acute heart failure), the question was asked of respondents from all relevant specialties. For each given care activity, the clinician was presented with the current distribution across sites of care and asked, “based on your clinical judgment, what portion of [care activity] could safely occur in each of the following sites of care, without compromising clinical outcomes?” Respondents were asked to recreate a 100% distribution across sites (including an “other” category) on what could be possible “with today’s technology” and “7-10 years from now, with expected advancements in technology.” Furthermore, each survey respondent was asked about 9 enablers and 4 barriers.

### Survey Administration

Full details of survey administration are available in the eMethods in Supplement 1. From September 17 to November 22, 2021, we surveyed 1069 practicing US clinicians in 21 specialties plus hospitalists. We used Intellisurvey to administer a web-based survey. Intellisurvey used multiple partners to field clinicians and ensured that we had a broad representation across all specialties of interest. All respondents voluntarily participated in this survey and provided consent for their responses to be used for research purposes and reported publicly in an aggregated and deidentified form. Samples were selected such that a minimum of 34 clinicians responded to each question. A total of 4608 respondents completed the screening questions for the survey, and 1069 respondents (23.2%) met the criteria to participate.

### Statistical Analysis

For each care activity, a mean unadjusted site-specific allowed payment amount was calculated based on 2019 claims data (eg, the allowed Medicare payment for arthroscopic knee surgery in an ASC or the allowed amount for the same procedure in the hospital inpatient setting). These amounts were calculated separately for the Medicare Limited Data Set and commercial claims from Merative given differences in payment structure and variability between the 2 insurance types. Professional and nonprofessional spending per procedure were calculated at the most granular level available in claims. Spending and volume of laboratory measures and images performed within the context of an admission (ie, diagnosis-related group) were included as part of the admission and not under the ancillary care activity type. In instances where no current data existed for a care activity at a site to which care was being shifted, we took the same proportional reduction in a specialty-specific care activity type (for example, anesthesiology evaluation and management [EM]) where available; if not available, we took the proportional reduction for the specialty-agnostic care activity type (eg, EM).

A straight mean of the survey responses was taken for each of 312 care activities to construct the new clinician-reported distribution. We ran χ^2^ tests to determine whether there were statistically significant differences between the original presented distribution and the new distribution for each care activity. In addition, we used a normal approximation to fit 95% CIs to the proportion of clinicians who felt that care activities (at a specialty and care activity type level) could move out of hospital-based settings (defined as hospital inpatient, hospital outpatient, and emergency department). Total spending was then calculated under each period based on the mean payment per care activity per site. For commercial and Medicare spending, the savings rate was extrapolated to the remaining portion of spending by 0% to 50%. Given the absence of reliable national Medicaid data, Medicaid spending was assumed to be equivalent to the lower of the estimated Medicare or commercial savings rate.

A list of 9 enablers and 4 barriers to site-of-care shifts was developed based on presurvey interviews with clinicians and a literature review.^1,26,27^ Respondents were asked to select the top 3 most important enablers needed to shift more care to alternative sites (eg, home or virtual) and the top 3 greatest barriers. Participants were then also asked to rate their level of agreement with the following statement for each of their top 3 selected enablers: “I believe that [enabler] is fully developed and ready to reach the maximum potential for care shifts.”

We used R statistical software version 4.2.0 (R Project for Statistical Computing) for claims analysis and Excel software version 16.24.06.0013 (Microsoft Corp) to conduct all survey analyses. All inferences are based on 95% CIs based on surveyed physician opinion. Data were analyzed from April 2022 through October 2023.

## Results

### Characteristics of Survey Respondents and Baseline Site-of-Care Distributions

Survey respondents included 1069 practicing clinicians (386 female [36.1%]; mean [SD] years since residency of physicians, 21.0 [9.7] years; mean [SD] age of nonphysicians, 45.3 [9.4] years) across 21 specialties plus hospitalists, all of whom practiced more than 20 clinical hours per week. There were 794 physicians (74.3%), and the remaining 275 respondents (25.7%) were a mix of nurse practitioners, physician assistants, nurse anesthetists, radiology and imaging technicians, and psychologists (Table 1). Between 34 and 74 clinicians were surveyed for each specialty, with a minimum of 34 clinicians responding to each care-activity question, spread across clinician type, sex, employment, years practicing, and primary place of practice (eTables 1 and 2 in Supplement 2).

**Table 1.** Characteristics of Respondents

Characteristic	Respondents, No. (%) (N = 1069)
Demographics	
Clinician type	
Physicians	794 (74.3)
Nonphysicians	275 (25.7)
Sex	
Female	386 (36.1)
Male	683 (63.9)
Time since residency, mean (SD), y	21.0 (9.7)
Age, mean (SD), y	45.3 (9.4)
Specialty (physicians only)	
Anesthesiology	30 (3.8)
Cardiology	30 (3.8)
Cardiothoracic surgery	34 (4.3)
Dermatology	36 (4.5)
ENT or otolaryngology	35 (4.4)
Family medicine or internal medicine	33 (4.2)
Gastroenterology	31 (3.9)
General surgery	58 (7.3)
Hematology or oncology	41 (5.2)
Hospitalist	33 (4.2)
Neurology	30 (3.8)
Neurosurgery	37 (4.7)
OBGYN	32 (4.0)
Ophthalmology	42 (5.3)
Orthopedic surgery	41 (5.2)
Pain management	30 (3.8)
Pediatrics	32 (4.0)
Physiatry or rehabilitative medicine	33 (4.2)
Plastic surgery	33 (4.2)
Psychiatry or behavioral health	30 (3.8)
Radiology	63 (7.9)
Urology	30 (3.8)
Employment status	
Hospital owned or employed	464 (43.4)
Medical group employed	353 (33.0)
Other	252 (23.6)
Weekly hours	
20-40	323 (30.2)
41-60	587 (54.9)
≥61	159 (14.9)
Primary site	
Inpatient	225 (21.0)
Outpatient	118 (11.0)
Physician office	535 (50.0)
All other	191 (17.9)
Geography	
East North Central	168 (15.7)
East South Central	38 (3.6)
Middle Atlantic	198 (18.5)
Mountain	65 (6.1)
New England	54 (5.1)
Pacific	169 (15.8)
South Atlantic	222 (20.8)
West North Central	69 (6.5)
West South Central	86 (8.0)

Abbreviations: ENT, ear, nose, and throat; OBGYN, obstetrics and gynecology.

For the 312 care activities examined, hospital-based settings represented a mean (SD) of 33.4% (29.4%) of volume and 71.2% (36.6%) of spending at baseline for commercial settings and 38.4% (31.1%) of volume and 66.0% (40.0%) of spending at baseline for Medicare settings. Among care activity types, the mean (SD) volume of care provided in hospital-based settings ranged from 23.5% (24.3%) for EM to 98.5% (6.0%) for facility-based care in commercial settings and 25.1% (22.7%) for EM to 100% (1.0%) for facility-based care in Medicare settings. Among specialties, the mean (SD) volume of care provided in hospital-based settings ranged from 2.2% (12.4%) for pediatrics to 100% (0.3%) for neurosurgery in commercial settings and 0.6% (7.7%) for pediatrics to 100% (0.2%) for neurosurgery in Medicare settings. Relative to the original presented distribution, new distributions reported by clinicians were statistically significantly different at the 95% level for 239 of 312 care activities (76.6%) surveyed (eTables 3 and 4 in Supplement 2).

### Overall Outcomes

Among 312 care activities surveyed, respondents indicated that 10.3 percentage points (95% CI, 10.0-10.5 percentage points) of commercial and 10.9 percentage points (95% CI, 10.7-11.1 percentage points) of Medicare volume currently taking place in hospital-based settings could shift to alternative sites with today’s technology without compromising clinical outcomes (Figure; eTable 5 in Supplement 2). This equates to a 30.8% reduction in hospital-based care in commercial settings and a 28.4% reduction in Medicare settings. Volume increases would be seen across postacute care and rehabilitation (55.9-fold for commercial settings and 610.4-fold for Medicare settings), home and virtual (26.9-fold for commercial settings and 11.3-fold for Medicare settings), ancillary (4.8-fold for commercial settings and 2.2-fold for Medicare settings), ASCs (1.9-fold for commercial settings and 1.9-fold for Medicare settings), and community (1.0-fold for commercial settings and 1.2-fold for Medicare settings) (eTables 3 and 4 in Supplement 2).

**Figure.**
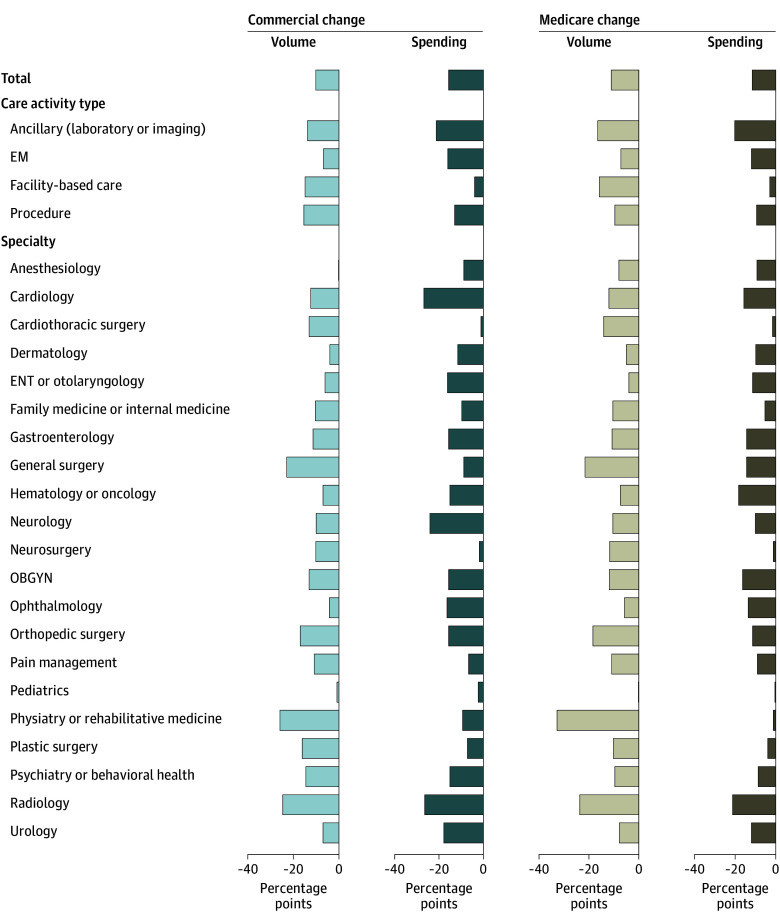
Change in Volume and Spending in Hospital-Based Settings Changes are shown by insurance type using today's technology. EM indicates evaluation and management; ENT, ear, nose, and throat; OBGYN, obstetrics and gynecology.

Translating this to net savings, we estimated that, with today’s technology, these site-of-care shifts could be associated with annual net savings of 5.2% for commercial and 8.9% for Medicare settings (eTables 7 and 8 in Supplement 2). Across the entire US health care system, this could be associated with reductions in overall 2019 US health care consumption spending ($3 562 339 000 000 000) by $113 767 446 087 174 (3.2%) to $147 661 672 284 263 (4.1%) annually.

Over a 7- to 10-year period with assumed technology advancements, respondents indicated 13.9 percentage points (95% CI, 13.5-14.2 percentage points) of commercial and 15.1 percentage points (95% CI, 14.8-15.3 percentage points) of Medicare volume, or 10.2% and 13.9% net savings respectively, could shift from hospital-based settings (eTables 7 and 8 in Supplement 2). Across the entire US health care system, this could be associated with reductions in overall 2019 US health care consumption spending ($3 562 339 000 000 000) by $208 140 011 801 182 (5.8%) to $303 198 368 922 344 (8.5%) annually.

### Site-of-Care Shifts by Care Activity Type

The volume shifting from hospital-based settings with today’s technology varied by care activity type. In commercial settings, EM visits would see the lowest volume shift (current state of 23.5% in hospital-based settings shifting to 16.6%, or a 6.9 percentage point change) and procedures would see the greatest volume shift (current state of 55.6% shifting to 39.9%, or a 15.7 percentage point change). In Medicare settings, EM visits would also see the lowest volume shift (current state of 25.1% shifting to 18.0%, or a 7.1 percentage point change), but ancillary (ie, laboratory measures and imaging) would see the greatest volume shift (current state of 46.8% shifting to 30.5%, or a 16.3 percentage point change) (Figure; eTables 5 and 7 in Supplement 2).

These rankings were the same for savings, where net savings for EM would be 2.0% for Medicare but an increase in spending of 1.8% in commercial settings. This occurred because observed commercial rates for some EM care activities were higher for the new site compared with the original site. Savings for facility-based care would be 13.1% in commercial and 15.2% in Medicare settings (eTables 5 and 7 in Supplement 2).

### Site-of-Care Shifts by Specialty

The volume shifts from hospital-based settings with today’s technology varied by specialty as well. Specialties with the greatest potential were physiatry or rehabilitative medicine (commercial: current state of 71.9% in hospital-based settings shifting to 45.6%, or a 26.3 percentage point change; Medicare: current state of 86.3% shifting to 54.0%, or a 32.3 percentage point change), radiology (commercial: current state of 67.4% in hospital-based settings shifting to 42.5%, or a 25.0 percentage point change; Medicare: current state of 70.0% shifting to 46.6%, or a 23.4 percentage point change), general surgery (commercial: current state of 86.5% in hospital-based settings shifting to 63.2%, or a 23.3 percentage point change; Medicare: current state of 85.3% shifting to 64.0%, or a 21.3 percentage point change), and orthopedic surgery (commercial: current state of 36.7% in hospital-based settings shifting to 19.5%, or a 17.2 percentage point change; Medicare: current state of 41.3% shifting to 23.1%, or an 18.2 percentage point change). Clinicians expected that even greater volume could be shifted without compromising clinical outcomes over 7 to 10 years (eg, a 35.9 percentage point change in commercial volume for general surgery vs a 23.3 percentage point change using today’s technology) (Figure; eTables 6 and 8 in Supplement 2).

Although volume shifts generally remained consistent between commercial and Medicare settings, the savings potential varied greatly, including an increase in spending for certain specialties based on 2019 observed commercial and Medicare rates (eg, dermatology, hematology or oncology, and pain management). This occurred because, for example, observed commercial rates for some dermatology procedures were higher for the new site compared with the original site. Specialties with the greatest net savings potential for commercial and Medicare settings respectively with today’s technology included physiatry or rehabilitative medicine (29.7% and 36.7%) and plastic surgery (23.3% and 24.3%). Double-digit savings were also estimated for many other surgical specialties (eg, cardiothoracic surgery; ear, nose, and throat or otolaryngology; general surgery; neurosurgery; and orthopedic surgery).

### Enablers and Barriers

Respondents most frequently selected the following enablers to shifting care to alternative sites: technological advancement, including remote patient monitoring or telemedicine (selected by 656 respondents [61.4%] in their top 3 enablers and 236 respondents [22.1%] as their top enabler); reimbursement models incentivizing the use of alternative sites (selected by 543 respondents [50.8%] in their top 3 and 201 respondents [18.8%] as their top enabler); and a perception of equivalent or superior quality of care at alternative sites (selected by 502 respondents [47.0%] in their top 3 and 216 respondents [20.2%] as their top enabler). Respondents did not believe that these enablers are fully developed today. A total of 33 respondents (3.1%) said that it was “completely true” that technology was fully developed and ready to reach the maximum potential for site-of-care shifts; 32 respondents (3.0%) said that was the case for quality, and 24 respondents (2.2%) said that was the case for reimbursement. Among barriers, respondents ranked economic incentives, such as payment arrangements and ownership models, most frequently (selected by 926 respondents [86.6%] in their top 3 barriers and 393 respondents [36.8%] as their top barrier), followed by continuity of care (selected by 895 respondents [83.7%] in their top 3 and 351 respondents [32.8%] as their top barrier) (Table 2).

**Table 2.** Survey Respondent Views on Enablers and Barriers for Shifts to Alternative Sites

Factor	Respondents, No. (%)					
	All respondents (N = 1069)		Physicians (n = 794)		Nonphysicians (n = 275)	
	In top 1	In top 3	In top 1	In top 3	In top 1	In top 3
Enabler						
Availability	94 (8.8)	353 (33.0)	61 (7.7)	249 (31.4)	33 (12.0)	104 (37.8)
Awareness	83 (7.8)	232 (21.7)	59 (7.4)	155 (19.5)	24 (8.7)	77 (28.0)
Convenience	95 (8.9)	309 (28.9)	76 (9.6)	239 (30.1)	19 (6.9)	70 (25.5)
EHR integration	61 (5.7)	221 (20.7)	50 (6.3)	166 (20.9)	11 (4.0)	55 (20.0)
Patient out-of-pocket costs	52 (4.9)	224 (21.0)	36 (4.5)	158 (19.9)	16 (5.8)	66 (24.0)
Quality	216 (20.2)	502 (47.0)	153 (19.3)	378 (47.6)	63 (22.9)	124 (45.1)
Reimbursement	201 (18.8)	543 (50.8)	153 (19.3)	424 (53.4)	48 (17.5)	119 (43.3)
Technology	236 (22.1)	656 (61.4)	185 (23.3)	485 (61.1)	51 (18.5)	171 (62.2)
Workflow integration	25 (2.3)	153 (14.3)	17 (2.1)	118 (14.9)	8 (2.9)	35 (12.7)
Other	6 (0.6)	14 (1.3)	4 (0.5)	10 (1.3)	2 (0.7)	4 (1.5)
Barrier						
Certificate of need	152 (14.2)	553 (51.7)	125 (15.7)	414 (52.1)	27 (9.8)	139 (50.5)
Clinician privileges or affiliation	164 (15.3)	798 (74.6)	113 (14.2)	594 (74.8)	51 (18.5)	204 (74.2)
Continuity of care	351 (32.8)	895 (83.7)	253 (31.9)	657 (82.7)	98 (35.6)	238 (86.5)
Economic incentives	393 (36.8)	926 (86.6)	294 (37.0)	694 (87.4)	99 (36.0)	232 (84.4)
Other	9 (0.8)	35 (3.3)	9 (1.1)	23 (2.9)	0 (0.0)	12 (4.4)

Abbreviation: EHR, electronic health record.

## Discussion

In this survey study based on the perspectives of 1069 clinicians focused on 312 care activities, we estimated that more than a quarter of volume (eg, procedures and diagnostics) could potentially shift to nonhospital sites with today’s technology without compromising clinical outcomes. The potential for shifts in the site of care delivery varied by specialty, with little of emergency clinician activities shifting but a large proportion of activities within physical medicine and rehabilitation doing so.

Extrapolating our results across the entire US health care system, we estimated a net savings opportunity of $113.8 billion to $147.7 billion (3.2%-4.1%) using today’s technology. This estimate increased to $208.1 billion to $303.2 billion (5.8%-8.5%) over 7 to 10 years with expected advancements in technology that have the potential to make these site-of-care shifts easier and safer.

However, there are barriers to realizing these savings. One major barrier is economic incentives, the most commonly cited barrier by survey respondents. Shifts from hospital-based settings would meaningfully decrease inpatient revenue, which may pose challenges for many established health systems operating on relatively thin margins.^28^ Furthermore, health systems often have large capital investments in hospital-based settings and little incentive to reduce revenue associated with those investments. Value-based and alternative payment models that move progressively toward more population-based payments could provide a degree of financial flexibility for health systems to innovate in this space but will likely need to represent a substantial proportion of their income statements before this meaningfully changes their incentives.

There are also important issues of capacity. For example, our results suggest that commercial and Medicare volume in ASCs could increase by more than 85%; this would clearly not be achievable without additional investment in physical and personnel resources in ASCs. Furthermore, as these sites expand, there is a risk of exacerbating existing inequities in care access. Research has shown that populations at increased risk have less access to ASCs, urgent care clinics, and high-quality home health agencies.^29,30,31,32,33,34^

Another important barrier is clinical inertia, driven by a combination of habit and a lack of knowledge about potentially different ways of doing things. One possibility is that shifts could be encouraged through nudges.^35^ Examples may include changing a default site of care for a colonoscopy from the hospital to an outpatient center based on a reordering of options presented to a scheduler. Furthermore, clinicians may need greater education about and awareness of eligibility criteria for care activities that are suitable for alternative sites. Increased electronic health record integration or interoperability would also be important to ensure continuity of patient care between sites. Expanding clinician access to the full range of practice sites through privileges, formal partnerships, or joint ventures could also lower barriers to using alternative sites.

There are reasons to believe these barriers can be overcome. The COVID-19 pandemic and resulting public health emergency and legislation enacted by Congress accelerated regulatory changes that have increased the use of alternative sites. Examples include the Acute Hospital Care at Home waiver, which had close to 300 hospital participants by 2023 and allows Medicare-certified hospitals to treat inpatient-level patients at home for more than 60 conditions, and the expansion of telehealth usage in Medicare. Both of these are currently in place through 2024.^36,37,38,39^ Other changes, such as site-neutral payments, the reduction of procedures on Medicare’s “inpatient only” list, and the expansion of services included on the ASC-covered payable list under Medicare, have also contributed to the increasing use of alternative sites.^40^

Our findings should be considered in the context of the existing literature. To date, these studies are limited in their generalizability because they often represent gross savings not net savings and examine 1 site at a time vs holistically considering all potential sites where care could shift.^1,2,3,4,5,6,7,8,9,10,11^ Our research expands on existing knowledge by estimating volume shifts and net savings across various specialties and care activity types based on clinician perspectives about site-of-care shifts.

### Limitations

Our study has limitations. Estimates were based on clinician assessment using a survey mechanism that has a relatively small sample size for each specialty. Clinicians were asked to opine on care activities that may be beyond the scope of their individual subspecialty practices; for example, orthopedic spine and orthopedic foot and ankle subspecialists have different degrees of familiarity with orthopedic procedures outside of their subspecialty. Furthermore, sample sizes were not sufficient to evaluate correlations between clinician characteristics that may have altered their willingness to recommend an alternative site.

Additionally, we surveyed respondents about 312 care activities that represent volumes of 1366.8 million commercial activities (48.8%) and 930.8 million Medicare activities (49.6%) in our claims sample. There is a long tail of care activities with relatively low volume that were not feasible to survey comprehensively and may have usage patterns that are different from what we observed.

For spending, we assumed all allowed amounts would stay the same, but payers may raise or lower rates as volume shifts, and any new site-neutral payment policies could also impact our estimates. In reality, these changes could go in either direction based on a trade-off between incentivizing shifting and preserving profits. In addition, our analysis used 2019 claims to avoid bias from temporary disruptions in care patterns resulting from the COVID-19 pandemic. The pandemic likely caused changes to site-of-care patterns that were not captured in our claims-based baseline distributions. However, given that our survey was conducted in 2021, our new clinician-based distributions should reflect a post–COVID-19 mindset in thinking about care patterns. In addition, we acknowledge that our savings estimates are reflective of the mix of payment models as represented in 2019 claims; as payment models evolve (such as through the take-up of value-based care), our savings estimates would change.

## Conclusions

This survey study examined 312 care activities, and clinician estimates suggested that existing technology could be used to shift more than a quarter of current volume away from hospital-based settings without compromising clinical outcomes. For the overall US health care system, this could be associated with annual savings of $113.8 billion (3.2%) to $147.7 billion (4.1%) based on observed rates. These findings may have broad implications for clinicians, organizations, and policymakers seeking to improve efficiency in health care delivery.

## Notes

Supplement 1.
eMethods.

Supplement 2. **eTable 1.** Characteristics of respondents by specialty **eTable 2.** Respondent specialty-level summary **eTable 3.** Care activity-level summary of volume and spend for 7 sites **eTable 4.** Specialty-level summary of volume and spend for 7 sites **eTable 5.** Change in volume and spending in hospital-based settings by care activity type by insurance type using today’s technology **eTable 6.** Change in volume and spending in hospital-based settings by specialty by insurance type using today's technology **eTable 7.** Estimated volume and spending in hospital-based settings by care activity type by insurance type across two time periods **eTable 8.** Estimated volume and spending in hospital-based settings by specialty by insurance type across two time periods

Supplement 3.
Data Sharing Statement
