# Supplementary material for: Potential US Health Care Savings Based on Clinician Views of Feasible Site-of-Care Shifts
Source: JAMA Netw Open. 2024 Aug 14;7(8):e2426857. doi: 10.1001/jamanetworkopen.2024.26857 (PMC11325203; doi:10.1001/jamanetworkopen.2024.26857)
Supplement: Supplement 1. — eMethods. [file jamanetwopen-e2426857-s001.pdf]

Supplemental Online Content

Sahni NR, Marine C, Cutler DM, et al. Potential US health care savings based on clinician views of feasible site-of-care shifts. *JAMA Netw Open*. 2024;7(8):e2426857. doi:10.1001/jamanetworkopen.2024.26857

eMethods.

This supplemental material has been provided by the authors to give readers additional information about their work.

© 2024 Sahni NR et al. *JAMA Network Open.*

Claims datasets: We used two claims datasets for our analyses: Commercial data from Merative and the Medicare Limited Data Set. We selected 2019 to avoid irregularities due to the COVID-19 pandemic. The Merative data for 2019 covered approximately 357 million claims. The Medicare Limited Data Set covered approximately 252 million claims. All analyses were conducted in R.

Specialties: A multidisciplinary panel of 33 physicians reviewed the claims data and sorted more than 5,000 individual CPT, DRG, and ICD codes into one of 26 unique specialties that were deemed most likely responsible for that procedure or condition. When differing points-of-view arose, a small group of three physicians would collectively make the final decision. This same group reviewed every final decision.

Five specialties were dropped for low volume, leaving 21 specialties representing 94.1% of 2019 commercial spending and 94.8% of utilization volume, and 92.7% of 2019 Medicare spending and 91.3% of utilization volume in our claims sample. The specialties were: anesthesiology; cardiology; cardiothoracic surgery; dermatology; ear, nose, and throat or otolaryngology; family medicine or internal medicine; gastroenterology; general surgery; hematology or oncology; neurology; neurosurgery; OBGYN; ophthalmology; orthopedic surgery; pain management; pediatrics; physiatry or rehabilitative medicine; plastic surgery; psychiatry or behavioral health; radiology; and urology.

Sites of care: We developed a taxonomy for sites of care. Four factors were used to define these sites: level of acuity of services offered; reimbursement rate (for example, differential reimbursement rates for hospital inpatient and outpatient departments);

© 2024 Sahni NR et al. *JAMA Network Open.*

clinician presence (for example, urgent care clinics in Texas are legally required to staff at least one board-certified physician, while retail clinics allow the physician to delegate authority to physician assistants and advanced practice nurses); and national scope (for example, excluding freestanding emergency departments, since they are not licensed in all states).

This resulted in 16 distinct sites of care, which were used for the survey and analysis: ambulatory surgery centers, dialysis clinics, emergency departments, freestanding imaging centers, freestanding laboratories, home, hospital inpatient, hospital outpatient, infusion centers, post-acute care/rehab centers, physician's office, retail clinic, urgent care center, and virtual. When reporting our results, these were grouped into seven types of sites: hospital-based settings (hospital inpatient, hospital outpatient, and emergency departments); post-acute facilities (inpatient rehabilitation, long-term acute care, and skilled nursing facilities); physicians' offices; community facilities (urgent care clinics and retail clinics); ambulatory surgery centers (ASCs); ancillary service sites (freestanding imaging centers, freestanding laboratories, dialysis centers, and infusion centers); and home and virtual (in-home care and virtual care).

Care activities: From the initial 5,000 candidate items, 312 unique care activities were prioritized for the survey. These accounted for 54.8% of total commercial claims spending, and 46.8% of Medicare spending in our claims sample. These percentages varied by specialty; for example, 80.2% of commercial dermatology spending was covered, compared with 43.1% of commercial OBGYN spending.

© 2024 Sahni NR et al. *JAMA Network Open.*

Care activities were categorized into four types: facility-based care such as management of acute pneumonia or heart failure; procedures such as cardiac bypass surgery or a breast biopsy; evaluation and management (EM) visits such as a pre-operative visit or a new patient visit with physical exam; and ancillary services, which included labs such as STD testing on blood, urine, and genital specimens or genetic testing for cancer for non-hospitalized, non-emergency patients and imaging such as a CT of the spine for non-admitted patients or a MRI of the brain for non-hospitalized, non-emergency patients.

Individual CPT and DRG descriptors were grouped in a way that respondents would easily understand. For example, “percutaneous coronary intervention,” “other coronary angiography,” “percutaneous coronary intervention (PCI) and stent placement,” and “PCI intervention, other or unspecified” were all tied to the care activity: “Cardiac catheterization including coronary angiogram and stent placement (PCI).” Spending and volume of labs and images performed within the context of an admission (i.e., DRG) were included as part of the admission, and not under “ancillary.” Observation encounters and emergency department visits were asked about separately by specialty, and all were grouped under “facility-based care.” Similarly, anesthesia for specific procedures was grouped with the procedure, and for each procedure relevant respondents were asked to determine whether that procedure could safely take place in the context of an inpatient admission, in a hospital-based outpatient department (which would still be categorized as facility-based in the aggregate responses), an ambulatory surgery center, a physician office, or the patient’s home.

© 2024 Sahni NR et al. *JAMA Network Open.*

Creating distributions: For each care activity, an original site of care distribution was created for the survey. This was developed directly from the claims data for commercial and Medicare. The same panel of 33 physicians reviewed each of these distributions to ensure they represented expected reality to avoid any data irregularity issues.

Enablers and barriers: A list of enablers and barriers was developed based on pre-survey interviews with clinicians and a literature review. Nine enablers were included in the survey: availability of alternative sites; physician and patient awareness of alternative sites and their capabilities; convenience of giving or receiving care at alternative sites; the integration of alternative sites with electronic health records; patient out-of-pocket costs incentivizing the use of alternative sites; perceived equivalency of quality of care delivered at alternative sites; favorable reimbursement at alternative sites; technological advancement; and the integration of alternative sites with existing provider workflows and clinical protocols. Four barriers were included: certificate of need laws restricting the development of alternative sites; perceived loss of continuity of care at alternative sites; limited clinician privileges or affiliations with alternative sites; and economic incentives (such as payment arrangements and ownership models).

Survey administration: A total of 4,608 respondents completed the screening questions for the survey, and 1,069 respondents (23.2%) met the criteria to participate. A total of 1,783 respondents (38.7%) were screened out because the desired number of respondents with their specialties had already been attained; 949 respondents (20.6%) were removed from the pool because they did not pass one of the other screening

© 2024 Sahni NR et al. *JAMA Network Open.*

questions (for example, not currently practicing or practicing fewer than 20 hours per week). A total of 807 respondents (17.5%) were excluded from the results for other factors (for example, failing “sense check” questions, being inactive after initiating the survey).

All respondents practiced more than 20 clinical hours per week. To ensure substantial input from non-physician clinicians, the number of clinicians in certain specialties was increased (for example, for cardiology, 26.8% of the 41 respondents were non-physician clinicians). Physicians who had completed residency before 1980 or after 2019 and non-physician clinicians under the age of 25 or over the age of 80 were excluded.

Survey questions: Any respondent completing the full survey answered a total of 6 to 21 questions related to care activities, depending on the respondent’s specialty. For two specialties with more than 20 care activities (general surgery, which had 41, and radiology, with 27), additional survey respondents were recruited, and respondents were randomized to receive only half of their specialty’s care-activity questions.

Each of the 312 survey questions were asked in the format below.

© 2024 Sahni NR et al. *JAMA Network Open.*

Based on your clinical judgment, what portion of **Cardiac pacemaker or defbrillator (ICD) placement** could safely occur in each of the following sites of care, without compromising clinical outcomes?

While answering this question, please assume that:

- The least acute sites of care should be used to their MAXIMUM POTENTIAL ignoring potential constraints (e.g., state regulations, patient adoption, reimbursement), which will be discussed later
- Patient acuity mix reflects the full spectrum that exists for this condition today, with the current state site of care breakdowns (e.g., if 100% inpatient today, then this represents the total days of all hospital admissions for the condition that occur today; if 100% emergency department today then this represents all patients presentations to the ED today)
- A portion of the patient care journey may be able to shift (e.g., early discharge to a post-acute site or home health; virtual physician rounds while a patient is hospitalized)

Percentages can be rough approximates and should sum to 100%.

Please do not exceed current state % in highest acuity (top listed) site. Please do not enter values lower than the current state % in lowest acuity (bottom listed) site.

	Current state (for reference)	With today's technology	7-10 years from now, with expected advancements in technology
Hospital inpatient	90-100%	%	%
Emergency department	0%	%	%
PAC / rehab (includes Skilled Nursing Facilities, long-term acute care, and IRFs)	0%	%	%
Ambulatory surgery center	0%	%	%
Physician's office	0%	%	%
Freestanding laboratory	0%	%	%
Home	0%	%	%
Virtual ⓘ	N/A	%	%
Other Specify		%	%
Total:	100%	0 %	0 %

Based on your clinical judgment, what portion of **Removal of ovary and tubes** could safely occur in each of the following sites of care, without compromising clinical outcomes?

While answering this question, please assume that:

- The least acute sites of care should be used to their MAXIMUM POTENTIAL ignoring potential constraints (e.g., state regulations, patient adoption, reimbursement), which will be discussed later
- Patient acuity mix reflects the full spectrum that exists for this condition today, with the current state site of care breakdowns (e.g., if 100% inpatient today, then this represents the total days of all hospital admissions for the condition that occur today; if 100% emergency department today then this represents all patients presentations to the ED today)
- A portion of the patient care journey may be able to shift (e.g., early discharge to a post-acute site or home health; virtual physician rounds while a patient is hospitalized)

Percentages can be rough approximates and should sum to 100%.

Please do not exceed current state % in highest acuity (top listed) site. Please do not enter values lower than the current state % in lowest acuity (bottom listed) site.

	Current state (for reference)	With today's technology	7-10 years from now, with expected advancements in technology
Hospital inpatient	30%	%	%
Hospital-based outpatient surgery	63%	%	%
Ambulatory surgery center	7%	%	%
Home	0%	%	%
Other Specify		%	%
Total:	100%	0 %	0 %

Enablers and barriers were asked in the format below, with illustrations of how respondents would select their top 3 in order.

© 2024 Sahni NR et al. *JAMA Network Open.*

Please select the top three most important enablers that are needed in order to shift more care to lower acuity sites (e.g., outpatient, home, virtual)

**Reimbursement**: Provider reimbursement is sufficient enough to incentivize use of lower acuity / less intensive sites of care, including value-based reimbursement models (e.g., capitation, bundled payments, ACOs)	**Availability**: There are a sufficient number of physical locations to promote use of alternative sites of care (e.g., sufficient ambulatory surgery center capacity)	**Quality**: Physicians and/or patients perceive equivalent or superior quality of care vs. acute sites
**EHR integration**: Alternative sites are seamlessly integrated into existing EHR, making it easy to manage patient care and data across sites	**Workflow integration**: Alternative sites are clearly defined in provider workflows and clinical protocols	**Convenience**: It is easier to receive and deliver care outside of acute settings
**Awareness**: Physicians and/or patients are familiar with and aware of alternative sites and their capabilities	**Patients' out-of-pocket costs**: Insurance products incentivize alternative site of care use (e.g., in-network reimbursement / coverage, different co-pay amounts)	**Technology**: Physicians and patients have sufficient access to the technologies needed to enable care delivery in alternative sites (e.g., remote patient monitoring, telemedicine, remote surgery)
Other Specify		

Please select the top three most important enablers that are needed in order to shift more care to lower acuity sites (e.g., outpatient, home, virtual)

**Reimbursement**: Provider reimbursement is sufficient enough to incentivize use of lower acuity / less intensive sites of care, including value-based reimbursement models (e.g., capitation, bundled payments, ACOs)	**Availability**: There are a sufficient number of physical locations to promote use of alternative sites of care (e.g., sufficient ambulatory surgery center capacity)	**Quality**: Physicians and/or patients perceive equivalent or superior quality of care vs. acute sites
**EHR integration**: Alternative sites are seamlessly integrated into existing EHR, making it easy to manage patient care and data across sites	1 **Workflow integration: Alternative sites are clearly defined in provider workflows and clinical protocols**	**Convenience**: It is easier to receive and deliver care outside of acute settings
**Awareness**: Physicians and/or patients are familiar with and aware of alternative sites and their capabilities	**Patients' out-of-pocket costs**: Insurance products incentivize alternative site of care use (e.g., in-network reimbursement / coverage, different co-pay amounts)	**Technology**: Physicians and patients have sufficient access to the technologies needed to enable care delivery in alternative sites (e.g., remote patient monitoring, telemedicine, remote surgery)
Other Specify		

© 2024 Sahni NR et al. *JAMA Network Open.*

Please select the top three most important enablers that are needed in order to shift more care to lower acuity sites (e.g., outpatient, home, virtual)

**Reimbursement**: Provider reimbursement is sufficient enough to incentivize use of lower acuity / less intensive sites of care, including value-based reimbursement models (e.g., capitation, bundled payments, ACOs) 2	**Availability**: There are a sufficient number of physical locations to promote use of alternative sites of care (e.g., sufficient ambulatory surgery center capacity)	**Quality**: Physicians and/or patients perceive equivalent or superior quality of care vs. acute sites 3
**EHR integration**: Alternative sites are seamlessly integrated into existing EHR, making it easy to manage patient care and data across sites	**Workflow integration**: Alternative sites are clearly defined in provider workflows and clinical protocols	**Convenience**: It is easier to receive and deliver care outside of acute settings
**Awareness**: Physicians and/or patients are familiar with and aware of alternative sites and their capabilities	**Patients' out-of-pocket costs**: Insurance products incentivize alternative site of care use (e.g., in-network reimbursement / coverage, different co-pay amounts)	**Technology**: Physicians and patients have sufficient access to the technologies needed to enable care delivery in alternative sites (e.g., remote patient monitoring, telemedicine, remote surgery) 1
Other Specify		

Please rate your level of agreement with the following statement about enablers today:

I believe that **Reimbursement: Provider reimbursement is sufficient enough to incentivize use of lower acuity / less intensive sites of care, including value-based reimbursement models (e.g., capitation, bundled payments, ACOs)** is fully developed and ready to reach the maximum potential for care shifts.

Not at all true today – needs to be developed to maximize the potential for care shifts	Somewhat true today	Moderately true today	Completely true today – we are ready to reach the maximum potential for care shifts

© 2024 Sahni NR et al. *JAMA Network Open.*
