# Supplementary material for: Potential US Health Care Savings Based on Clinician Views of Feasible Site-of-Care Shifts
Source: JAMA Netw Open. 2024 Aug 14;7(8):e2426857. doi: 10.1001/jamanetworkopen.2024.26857 (PMC11325203; doi:10.1001/jamanetworkopen.2024.26857)
Supplement: Supplement 3. — Data Sharing Statement [file jamanetwopen-e2426857-s003.pdf]

Data Sharing Statement

Sahni. Potential US Health Care Savings Based on Clinician Views of Feasible Site-of-Care Shifts. *JAMA Netw Open*. Published online August 14, 2024. doi:10.1001/jamanetworkopen.2024.26857

Data
Data available: No

Additional Information
Explanation for why data not available: We informed survey respondents their data would be kept anonymous and only reported in the aggregate.
